# The impact of transformational leadership on nurses’ work alienation: the chain mediation of organizational climate and psychological empowerment

**DOI:** 10.3389/fpsyg.2026.1883672

**Published:** 2026-08-17

**Authors:** Kailin Yin, Manman Shi, Lin Ji, Jie Sun, Binbin Zhang

**Affiliations:** 1 School of Nursing, Shandong Second Medical University, Weifang, China; 2 School of Nursing and Rehabilitation, Cheeloo College of Medicine, Shandong University, Jinan, China; 3 Department of General Surgery, Qilu Hospital of Shandong University, Jinan, China

**Keywords:** chain mediation, nurses, organizational climate, psychological empowerment, transformational leadership, work alienation

## Abstract

**Aims:**

This study, based on social exchange theory, social cognitive theory, and self-determination theory, investigates the link between transformational leadership and nurses’ work alienation. It also examines if organizational climate and psychological empowerment mediate this relationship.

**Design:**

A cross-sectional correlational study.

**Methods:**

Between February and April 2026, researchers recruited 1,154 nurses from six hospitals in Shandong Province, China, using convenience sampling. They collected data with validated scales assessing transformational leadership, organizational climate, psychological empowerment, and work alienation. The analysis involved descriptive statistics, correlation analyses, and serial mediation analyses, conducted using SPSS 26.0 and Model 6 of the PROCESS macro (version 4.1).

**Results:**

Nurses experienced a moderate level of work alienation. There was a negative correlation between transformational leadership and work alienation. Both organizational climate and psychological empowerment mediated this relationship individually. Additionally, they acted together as a chain mediator between transformational leadership and work alienation.

**Conclusion:**

Transformational leadership had a direct negative relationship with work alienation. Additionally, it was indirectly linked to work alienation through the sequential mediation of organizational climate and psychological empowerment.

**Implications:**

The findings indicate that nursing managers should adopt transformational leadership behaviors, foster a supportive organizational climate, and enhance nurses’ psychological empowerment to reduce work alienation. These integrated strategies can guide leadership development programs and management practices to improve nurses’ occupational well-being and retention.

## 1 Introduction

The growing and aging global population intensifies the demand for healthcare services, underscoring the essential role of nurses in achieving universal health coverage, the Sustainable Development Goals, and enhanced population health outcomes (World Health Organization, 2021). However, a major challenge facing global health systems is the shortage of nurses (Estephan et al., 2023). This disparity between the limited supply of nurses and the increasing demand for quality care creates high-stress environments and heavy workloads, leaving nurses especially susceptible to work alienation.

Work alienation occurs when individuals become disengaged and psychologically detached from their work due to unmet material and spiritual needs (Amarat et al., 2019). Recently, there has been a growing focus on work alienation among nurses. This negative psychological state emerges when nurses’ needs or expectations do not align with their work, resulting in feelings of helplessness, powerlessness, and meaninglessness (Li et al., 2023). Research shows that Chinese nurses experience moderate to high levels of work alienation, a particularly concerning issue (You et al., 2022). High alienation levels correlate with reduced work engagement (Kartal, 2018), lower job satisfaction (Chiaburu et al., 2013), and decreased quality of nursing care (Eskin Bacaksiz et al., 2020). Additionally, increased alienation is linked to higher turnover intentions, potentially worsening the existing nursing shortage (You et al., 2022). Therefore, identifying factors that influence nurses’ work alienation and implementing effective strategies to mitigate it is essential for advancing the nursing profession.

Nursing managers play a vital role in developing nursing teams, maintaining nurses’ health, and ensuring efficient nursing services (García et al., 2020). Leadership has emerged as a key component in nursing management (García-Sierra and Fernández-Castro, 2018). It involves guiding group activities toward a shared goal (Al-Sawai, 2013). The leadership of nursing managers influences nurses’ well-being (Sabbah et al., 2020), job satisfaction (Specchia et al., 2021), work engagement (Alluhaybi et al., 2024), nursing performance (Al-Rjoub et al., 2024) and reduces turnover intention (Cho and Steege, 2025). Recent leadership research highlights the limitations of motivation based solely on transactional relationships, prompting a shift toward transformational leadership. Transformational leaders elevate both leaders and employees to higher pursuits, focusing on self-development and intrinsic motivation rather than material rewards. They foster an atmosphere of trust, mobilize enthusiasm, encourage collective interests, and achieve goals beyond expectations (Bass, 1995). Studies indicate that transformational leadership negatively correlates with work alienation (Sarros et al., 2002). However, the link between head nurses’ transformational leadership and nurses’ work alienation, along with its mechanisms, remains underexplored. Additionally, organizational climate and psychological empowerment have been linked to nurses’ work alienation (Lee et al., 2022; Wang et al., 2025; Zheng et al., 2026). Few studies have examined how these factors mediate the relationship between transformational leadership and work alienation. This study aimed to explore the connection between transformational leadership and nurses’ work alienation and to verify the mediating roles of organizational climate and psychological empowerment.

## 2 Literature review and research framework

### 2.1 Theoretical framework

This study’s conceptual framework is based on three complementary theories. First, social exchange theory (Cropanzano and Mitchell, 2005) posits that workplace relationships are exchange processes. Employees reciprocate support and resources from leaders, explaining why transformational leadership can lead to positive employee responses and reduced work alienation. Second, social cognitive theory (Bandura, 1986) suggests a triadic reciprocal causation among the environment, individual, and behavior. Environmental cues influence individuals’ cognitive appraisals, guiding their emotional and behavioral responses. This theory highlights organizational climate as a crucial environmental mechanism for transmitting leadership effects. Third, self-determination theory (Ryan and Deci, 2000) asserts that supportive environments satisfy basic psychological needs for autonomy, competence, and relatedness, fostering motivation internalization. This theory underpins the idea that psychological empowerment is a motivational state influenced by organizational climate. Collectively, these theories outline a sequential mechanism: transformational leadership, as a high-quality exchange process, shapes nurses’ perceptions of organizational climate, enhancing psychological empowerment and ultimately reducing work alienation.

### 2.2 Transformational leadership and work alienation

Transformational leadership includes four key dimensions: idealized influence, inspirational motivation, intellectual stimulation, and individualized consideration (Lee and Shi, 2005). Together, these elements create a work environment marked by trust, empowerment, and shared values (Raja et al., 2018). According to social exchange theory (Cropanzano and Mitchell, 2005), these leadership behaviors trigger a reciprocal process. When nurses perceive support and investment from their leaders, they often respond with increased commitment and align organizational goals with personal ones, reducing work alienation. Empirical evidence supports this theory. Sarros et al. (2002) found that transformational leadership correlates with lower work alienation and mitigates the negative effects of bureaucratic structures in a general workforce sample. In nursing, transformational leadership by head nurses is linked to positive outcomes like higher work engagement (Liu et al., 2025; Zhang et al., 2025). However, work alienation remains common among Chinese nurses (You et al., 2022). Studies vary in modeling this relationship. Some suggest transformational leadership directly affects employee attitudes, while others see its influence as indirect, mediated by factors like job stress (Li et al., 2023) or negative interpersonal experiences (Liu et al., 2025). Most evidence comes from general employee samples, with limited research on nurses, especially in China. This convergence of theory and findings suggests a negative relationship between transformational leadership and work alienation, yet inconsistencies and gaps necessitate further study in nursing contexts. Thus, we propose Hypothesis 1: Transformational leadership is negatively correlated with nurses’ work alienation.

### 2.3 The mediating role of organizational climate

Organizational climate refers to employees’ perceptions of their work environment and tasks, influencing individual behavior and psychological well-being (Bronkhorst et al., 2015; Neal et al., 2000; Sein Myint et al., 2021). According to social cognitive theory (Bandura, 1986), environmental cues impact cognitive appraisals, guiding emotional and behavioral responses. Empirical studies reveal two main evidence streams. First, transformational leaders create efficient, visionary, and mission-driven climates (Zuraik and Kelly, 2018), and head nurses’ transformational leadership has been linked to favorable ward climates (Zhang et al., 2025). Second, perceived climate affects nurses’ cognitive appraisals: supportive and fair climates enhance efficacy beliefs (Chen et al., 2020) and positive outcome expectancies (Hoegen et al., 2022). These appraisals reduce feelings of helplessness, powerlessness, and meaninglessness, key components of work alienation (Xiao and Zhang, 2019). Despite these findings, the role of organizational climate varies across studies. It has been modeled as an antecedent of job stress (Li et al., 2023), a mediator between leadership and work engagement (Zhang et al., 2025), and a contextual correlate of employee mental health (Bronkhorst et al., 2015). Integrating these streams suggests organizational climate plausibly transmits leadership effects on work alienation, though this mediating pathway is rarely tested directly. Thus, we propose Hypothesis 2: Organizational climate mediates the relationship between transformational leadership and nurses’ work alienation.

### 2.4 The mediating role of psychological empowerment

Psychological empowerment refers to an individual’s perception of their own empowerment, encompassing meaning, self-determination, self-efficacy, and impact (Spreitzer, 1995). Research consistently shows a significant correlation between leadership and psychological empowerment (Choi et al., 2016; Sahraei Beiranvand et al., 2021; Younas et al., 2023; Zhang et al., 2022). Specifically, transformational leadership positively correlates with employees’ empowerment by fostering internal evaluation (Cheng et al., 2023). Empowered employees often recognize their work’s significance and enjoy decision-making autonomy, which boosts intrinsic motivation and reduces negative psychological states like work alienation (Yuan, 2019; Zheng et al., 2026). Together, these findings suggest an indirect pathway from transformational leadership to reduced work alienation through psychological empowerment. However, most empowerment research focuses on positive outcomes such as job satisfaction, work engagement, and citizenship behavior (Ibrahim et al., 2024). The impact of empowerment on negative states like work alienation has received limited empirical attention, making existing findings less comparable. Thus, we propose Hypothesis 3: Psychological empowerment mediates the relationship between transformational leadership and work alienation.

### 2.5 The chain mediating role of organizational climate and psychological empowerment

A key question is how the two mediators, organizational climate and psychological empowerment, relate to each other. Numerous studies have established a strong connection between these two factors (Akkoç et al., 2022; Mok and Au-Yeung, 2002; Wang et al., 2024). According to self-determination theory (Li et al., 2023; Ryan and Deci, 2000), a supportive organizational climate helps internalize extrinsic values and rules. This process satisfies individuals’ needs for autonomy, competence, and relatedness, thereby enhancing psychological empowerment. The causal relationship between these constructs has been inconsistently conceptualized. Some studies view organizational climate as an environmental factor that fosters psychological empowerment (Mok and Au-Yeung, 2002; Wang et al., 2024). Others consider empowerment as an antecedent that influences organizational climate (Lu et al., 2025). Even when a climate–empowerment sequence is suggested, these constructs are often examined as parallel rather than sequential mediators. This leaves their chain-mediating role in the leadership–alienation relationship untested. In summary, while a negative association between transformational leadership and work alienation is documented, the sequential mechanism linking them is not well understood. By integrating social cognitive theory with self-determination theory, we propose that transformational leadership enhances psychological empowerment by fostering a positive organizational climate. This climate, in turn, is related to reduced work alienation. Therefore, we propose Hypothesis 4: Transformational leadership indirectly affects nurses’ work alienation through the chain-mediating roles of organizational climate and psychological empowerment.

This study examined the relationships among transformational leadership, organizational climate, psychological empowerment, and work alienation. The objective was to determine how transformational leadership influences nurses’ work alienation.

## 3 Materials and methods

### 3.1 Study design

This study employed a cross-sectional design.

### 3.2 Participants and sample size

Participants were recruited through convenience sampling from six hospitals in various cities across Shandong Province, China, between February and April 2026. These hospitals were chosen for two reasons: they are located in different cities, offering diverse departmental compositions and management practices, which enhances the sample’s heterogeneity and representativeness; additionally, their nursing departments had established collaborative relationships with the research team, facilitating survey administration. Inclusion criteria required participants to: (1) hold a nurse practitioner qualification certificate; (2) have at least 1 year of employment; (3) be actively engaged in clinical nursing work; (4) have clear consciousness with no history of mental disorders; and (5) consent to participate in the study. Exclusion criteria included: (1) being on outside training, sick leave, maternity leave, or otherwise absent from work during the survey period; and (2) being an intern nurse or trainee, meaning not a regular hospital employee.

The sample size was determined during the study design using G-Power Version 3.1.9.7 (Kuo et al., 2025). For linear multiple regression with R^2^ deviation from zero, assuming a medium effect size (*f*^2^ = 0.15), *α* = 0.05, power = 0.80, and 14 predictors, the minimum required sample size was 135. To account for a potential 10% dropout rate or incomplete responses, the recommended sample size increased to at least 150 participants. In practice, a much larger sample was recruited. Participants were drawn from six hospitals in different cities and various clinical departments across Shandong Province. This larger sample aimed to enhance the representativeness and generalizability of the findings. The actual sample consisted of 1,154 valid questionnaires, significantly exceeding the requirement and ensuring adequate statistical power for the mediation analysis.

### 3.3 Variables and measurements

#### 3.3.1 General information questionnaire

We gathered general information such as gender, age, marital status, years of work experience, highest education level, professional title, department, employment status, monthly income, number of night shifts per month, and whether the participant was a specialized nurse.

#### 3.3.2 Transformational leadership

The Transformational Leadership Questionnaire evaluated nurses’ perceptions of transformational leadership. Permission for its use was secured from the original authors via email/personal communication. Developed by Lee and Shi (2005), this scale is tailored to China’s national conditions and cultural nuances. It has been extensively used among Chinese nurses (Liu et al., 2025; Zhang et al., 2025). The questionnaire consists of 26 items divided into four dimensions: moral modeling (8 items), vision inspiration (6 items), individualized consideration (6 items), and leader charisma (6 items). Examples include “My leader is honest and incorruptible and never seeks personal gain” and “My leader is the first to endure hardship and the last to enjoy comfort.” Responses are recorded on a 5-point Likert scale, from 1 (strongly disagree) to 5 (strongly agree), with total scores ranging from 26 to 130. Higher scores reflect greater perceived leadership. In this study, the CFA confirmed the scale’s four-factor structure, showing satisfactory model fit indices (*χ*^2^/df = 2.94, CFI = 0.968, TLI = 0.965, RMSEA = 0.041, SRMR = 0.040). The Cronbach’s *α* coefficients were 0.924 for moral modeling, 0.889 for vision inspiration, 0.890 for individualized consideration, and 0.876 for leader charisma, with an overall α of 0.936. AVE values ranged from 0.538 to 0.604, and CR values ranged from 0.875 to 0.924. The Fornell-Larcker criterion confirmed adequate discriminant validity, as the square root of AVE for each factor surpassed its correlations with other factors.

#### 3.3.3 Organizational climate

The organizational climate assessment scale, developed by He et al. (2011), is widely utilized in Chinese research (Lu et al., 2025; Zhai et al., 2025). This scale includes 24 items divided into four dimensions: fair supportive behavior (10 items), intimacy and aggressive climate behavior (5 items), colleague behavior (5 items), and interpersonal climate (4 items). Sample items are “Nurses in my unit get along very well with one another during work hours” and “Nurses in my unit interact closely and care about each other.” Each item is rated on a 5-point Likert scale, from 1 (strongly disagree) to 5 (strongly agree), with total scores ranging from 24 to 120. Higher scores indicate a more favorable organizational climate. Permission to use the scale was obtained from the original author(s) via email or personal communication. In this study, the CFA confirmed the scale’s four-factor structure, showing satisfactory model fit indices (*χ*^2^/df = 2.79, CFI = 0.973, TLI = 0.970, RMSEA = 0.039, SRMR = 0.035). The Cronbach’s *α* coefficients were 0.923 for fair supportive behavior, 0.869 for colleague behavior, 0.912 for interpersonal climate, and 0.846 for intimacy and aggressive climate behavior, with an overall α of 0.941. AVE values ranged from 0.523 to 0.726, and CR values ranged from 0.845 to 0.924. The Fornell-Larcker criterion confirmed adequate discriminant validity, as the square root of AVE for each factor exceeded its correlations with other factors.

#### 3.3.4 Psychological empowerment

The Psychological Empowerment Scale, originally developed by Spreitzer (1995), was used to evaluate nurses’ psychological empowerment. This scale, translated and revised by Lee et al. (2006), has shown strong reliability and validity in various studies (Malak and Abu Safieh, 2022). It includes four dimensions: meaning, self-efficacy, self-determination, and impact, each with three items, totaling 12 items. Sample items are “The work I do is very important to me” and “My job activities are personally meaningful to me.” Responses are recorded on a 5-point Likert scale, ranging from 1 (strongly disagree) to 5 (strongly agree). Total scores range from 12 to 60, with higher scores indicating greater psychological empowerment. Permission to use the scale was obtained from the original author(s) via email/personal communication. In this study, the CFA confirmed the scale’s four-factor structure, showing satisfactory model fit indices (*χ*^2^/df = 3.08, CFI = 0.987, TLI = 0.982, RMSEA = 0.042, SRMR = 0.023). The Cronbach’s *α* coefficients for the subscales were 0.847 (meaning), 0.875 (self-determination), 0.841 (self-efficacy), and 0.855 (impact), with an overall Cronbach’s α of 0.909. The AVE values ranged from 0.638 to 0.701, and the CR values ranged from 0.841 to 0.875. The Fornell-Larcker criterion confirmed adequate discriminant validity, as the square root of AVE for each factor exceeded its correlations with other factors.

#### 3.3.5 Work alienation

Nurses’ work alienation was assessed using the Work Alienation Scale, developed by Chinese scholar Ren (2012), and it has seen extensive use in China (Zhang et al., 2023). This questionnaire includes 12 items divided into three dimensions: helplessness, hopelessness, and meaninglessness, with four items per dimension. Examples of items are “My supervisor’s work instructions sometimes leave me unsure of what to do” and “Promotion seems like a distant hope for me.” It uses a 5-point Likert scale from 1 (strongly disagree) to 5 (strongly agree), with total scores ranging from 12 to 60, where higher scores indicate greater work alienation. Permission to use this scale was obtained from the original author(s) via email/personal communication. In the current study, the CFA confirmed the three-factor structure of the scale, with satisfactory model fit indices (*χ*^2^/df = 2.75, CFI = 0.991, TLI = 0.989, RMSEA = 0.039, SRMR = 0.021). The Cronbach’s α coefficients for the three subscales were 0.892 (helplessness), 0.912 (hopelessness), and 0.902 (meaninglessness), with an overall Cronbach’s α of 0.934. The AVE values ranged from 0.674 to 0.723, and the CR values ranged from 0.892 to 0.913. The Fornell–Larcker criterion indicated adequate discriminant validity, as the square root of AVE for each factor exceeded its correlations with other factors.

### 3.4 Data collection

Data collection was conducted via the Wenjuanxing platform. After obtaining ethical approval from all participating hospitals and consent from their nursing departments, we created an electronic poster containing a link to the online survey. This link was sent to head nurses in each department, who then invited eligible nurses to participate based on the inclusion criteria. The first page of the questionnaire included a standardized introduction that outlined the study’s purpose and provided completion instructions. It stressed voluntary and anonymous participation, allowing respondents to withdraw at any time. To ensure completeness, all questions were mandatory, and the questionnaire could not be submitted if any question was left unanswered. To prevent duplicate submissions, each IP address was restricted to completing only one questionnaire. We identified patterned responses by screening for identical answers across all items, such as all ‘3’ or alternating patterns like ‘1,2,1,2…’. A 200-s threshold was set based on a pilot test (*n* = 50), which showed that the minimum reasonable completion time was approximately 180 s; thus, 200 s served as a conservative cutoff to exclude careless responders. Although survey links were distributed through head nurses, participants were informed that their responses were anonymous, non-participation would not affect their employment, and they could withdraw at any time. Head nurses were instructed not to monitor participation.

### 3.5 Data analysis

We used SPSS Statistics 26.0 (IBM Corp.) for data analysis. To check for common method bias, we conducted Harman’s single-factor test. Descriptive statistics summarized participants’ sociodemographic details and study variables, presenting categorical data as frequencies and percentages, and continuous data as means and standard deviations. Pearson correlation analysis examined relationships among the four variables. For chain mediation effect analysis, we employed Model 6 in the PROCESS 4.1 macro in SPSS, utilizing the bootstrap method with 5,000 samples and 95% bias-corrected confidence intervals. Control variables, identified through univariate analysis as significantly associated with work alienation (*p* < 0.05), were included as covariates in all regression equations of PROCESS Model 6. Results were considered significant if the 95% bootstrap confidence interval did not include zero. Before mediation analysis, we assessed the normality of all variables and checked for multicollinearity among predictors. Q–Q plots and histograms showed that all variables approximately followed a normal distribution. The bootstrap method in the PROCESS macro is robust against moderate normality violations (Hayes, 2018), especially with large samples (*N* = 1,154). Variance inflation factor (VIF) values were below 3.0, indicating no multicollinearity issues.

### 3.6 Ethical approval

The Ethics Committee of Qilu Hospital of Shandong University approved this study (Approval No. KYLL-2026-01-074). Participants gave informed consent and participated voluntarily. No identifying information was collected, and all data were securely stored.

## 4 Results

### 4.1 Assessment of common method bias

In this study, we used self-report questionnaires and minimized common method bias by ensuring anonymity and randomizing items. Harman’s single-factor test revealed 14 factors with eigenvalues greater than 1. The first factor explained 29.34% of the total variance, which is below the critical 40% threshold. This finding suggests that common method bias was not a significant concern.

### 4.2 Characteristics of the participants

A total of 1,229 questionnaires were collected. After excluding 75 due to patterned responses or completion times under 200 s, 1,154 valid questionnaires remained for analysis. Table 1 shows the baseline demographic characteristics of these participants. The majority were female (92.8%), married (80.2%), and aged 30–39 years (50.5%). Nurses with five or fewer years of experience, a master’s degree or higher, working in emergency or intensive care units, earning less than 5,000 RMB monthly, or having more than eight night shifts per month reported significantly higher levels of work alienation (*p* < 0.05).

**Table 1:** Basic characteristics of the participants in this study (*N* = 1,154).

Variable	*N*(%)	Work alienation	*t*/*F*	*P*
Age (year)			2.105	>0.05
≤29	259(22.4)	33.86 ± 12.40		
30 ~ 39	583(50.5)	33.70 ± 11.07		
40 ~ 49	257(22.3)	35.70 ± 11.17		
≥50	55(4.8)	33.05 ± 11.06		
Gender			−1.665	>0.05
Male	83(7.2)	32.14 ± 10.70		
Female	1,071(92.8)	34.31 ± 11.46		
Marital status			2.417	>0.05
Single	213(18.5)	32.74 ± 11.48		
Married	925(80.2)	34.52 ± 11.38		
Divorced/Widowed	16(1.4)	31.94 ± 11.93		
Working experiences (years)			3.855	<0.05
≤5	225(19.5)	35.64 ± 11.88		
6 ~ 10	195(16.9)	32.55 ± 10.69		
>10	734(63.6)	34.12 ± 11.42		
Education level			3.060	<0.05
College diploma	137(11.9)	32.82 ± 12.60		
Bachelor degree	885(76.7)	33.99 ± 10.81		
Master’s degree or above	132(11.4)	36.65 ± 13.63		
Professional title			2.599	>0.05
Junior nurse	103(8.9)	31.44 ± 11.79		
Senior nurse	287(24.9)	34.99 ± 11.38		
Nurse-in-charge	605(52.4)	34.07 ± 11.28		
Co-chief nurse or above	159(13.8)	34.71 ± 11.58		
Departments			3.824	<0.05
Internal Medicine	326(28.2)	32.99 ± 9.74		
Surgery	249(21.6)	33.19 ± 9.56		
Obstetrics/Gynecology	106(9.2)	34.88 ± 11.87		
Pediatrics	39(3.4)	35.87 ± 15.29		
Emergency/Intensive care unit	234(20.3)	36.81 ± 13.66		
Operating Room	86(7.5)	35.77 ± 13.12		
Others	114(9.9)	31.65 ± 10.14		
Employment status			2.553	>0.05
Staff with establishment	388(33.6)			
Personnel Agency	192(16.6)			
Contract-based	450(39.0)			
Labor Dispatch	124(10.7)			
Monthly Income			4.117	<0.05
<5,000	118(10.2)	36.78 ± 12.89		
5,000 ~ 10,000	866(75.0)	34.03 ± 11.32		
>10,000	170(14.7)	32.96 ± 10.57		
Night shift (per month)			3.382	<0.05
0	202(17.5)	32.55 ± 12.60		
1 ~ 4	358(31.0)	33.75 ± 10.38		
5 ~ 8	441(38.2)	34.25 ± 10.69		
>8	153(13.3)	36.92 ± 13.55		
Specialist Nurse			−0.317	>0.05
Yes	281(24.4)	33.96 ± 11.50		
No	873(75.6)	34.21 ± 11.40		

### 4.3 Correlation between work alienation and transformational leadership, organizational climate, and psychological empowerment

The average scores for work alienation, transformational leadership, organizational climate, and psychological empowerment were 34.15 ± 11.42, 89.43 ± 11.27, 79.84 ± 15.72, and 39.74 ± 10.11, respectively. Pearson correlation analysis revealed a negative correlation between nurses’ work alienation and transformational leadership, organizational climate, and psychological empowerment (*r* = −0.432, *p* < 0.001; *r* = −0.561, *p* < 0.001; *r* = −0.639, *p* < 0.001, respectively). Transformational leadership showed a positive correlation with both organizational climate and psychological empowerment (*r* = 0.427, *p* < 0.001; *r* = 0.483, *p* < 0.001, respectively). Additionally, organizational climate was positively correlated with psychological empowerment (*r* = 0.643, *p* < 0.001) (see Table 2).

**Table 2:** Pearson’s correlation analyses between work alienation and transformational leadership, organizational climate, and psychological empowerment (*N* = 1,154).

Variables	Mean (SD)	Work alienation	Transformational leadership	Organizational climate	Psychological empowerment
Work alienation	34.15 (11.42)	1			
Transformational leadership	89.43 (11.27)	−0.432^***^	1		
Organizational climate	79.84 (15.72)	−0.561^***^	0.427^***^	1	
Psychological empowerment	39.74 (10.11)	−0.639^***^	0.483^***^	0.643^***^	1

^***^p < 0.001.

### 4.4 Chain mediation analysis

In the study, Model 6 of the PROCESS macro (version 4.1) was employed to test the serial mediation model. Transformational leadership was the independent variable, and work alienation was the dependent variable. Organizational climate and psychological empowerment served as mediators. Control variables included work experience, education level, department, monthly income, and night shifts. All path coefficients reported are standardized (*β*). The results showed that transformational leadership was significantly and negatively associated with work alienation (*β* = −0.424, *p* < 0.001). It was also positively associated with both organizational climate and psychological empowerment (*β* = 0.422, *p* < 0.001; *β* = 0.255, *p* < 0.001, respectively). Furthermore, organizational climate positively correlated with psychological empowerment (*β* = 0.532, *p* < 0.001). Even when considering the mediating variables, transformational leadership remained significantly and negatively associated with work alienation (*β* = −0.124, *p* < 0.001). Both organizational climate and psychological empowerment also significantly and negatively correlated with work alienation (*β* = −0.226, *p* < 0.001; *β* = −0.426, *p* < 0.001, respectively). Detailed regression analysis results are presented in Table 3.

**Table 3:** Regression analysis of the relationship between transformational leadership and work alienation (*N* = 1,154).

Regression equation		Fitting index		Significance	
Outcomes	Predictors	*R* ^2^	*F*	*β*	*t*
Work Alienation		0.202	48.235		
	Transformational Leadership			−0.424	−16.014^***^
	Covariates^1^				
Organizational Climate		0.189	44.614		
	Transformational Leadership			0.422	15.802^***^
	Covariates^1^				
Psychological Empowerment		0.469	144.382		
	Transformational Leadership			0.255	10.706^***^
	Organizational Climate			0.532	22.238^***^
	Covariates^1^				
Work Alienation		0.464	124.064		
	Transformational Leadership			−0.124	−4.947^***^
	Organizational Climate			−0.226	−7.864^***^
	Psychological Empowerment			−0.426	−14.371^***^
	Covariates^1^				

All reported path coefficients are standardized (β).

^1^Demographic variables (working experience, education level, department, monthly income, and night shift) were entered as covariates using the sequential approach in the PROCESS macro.

^***^p < 0.001.

**Figure 1:**
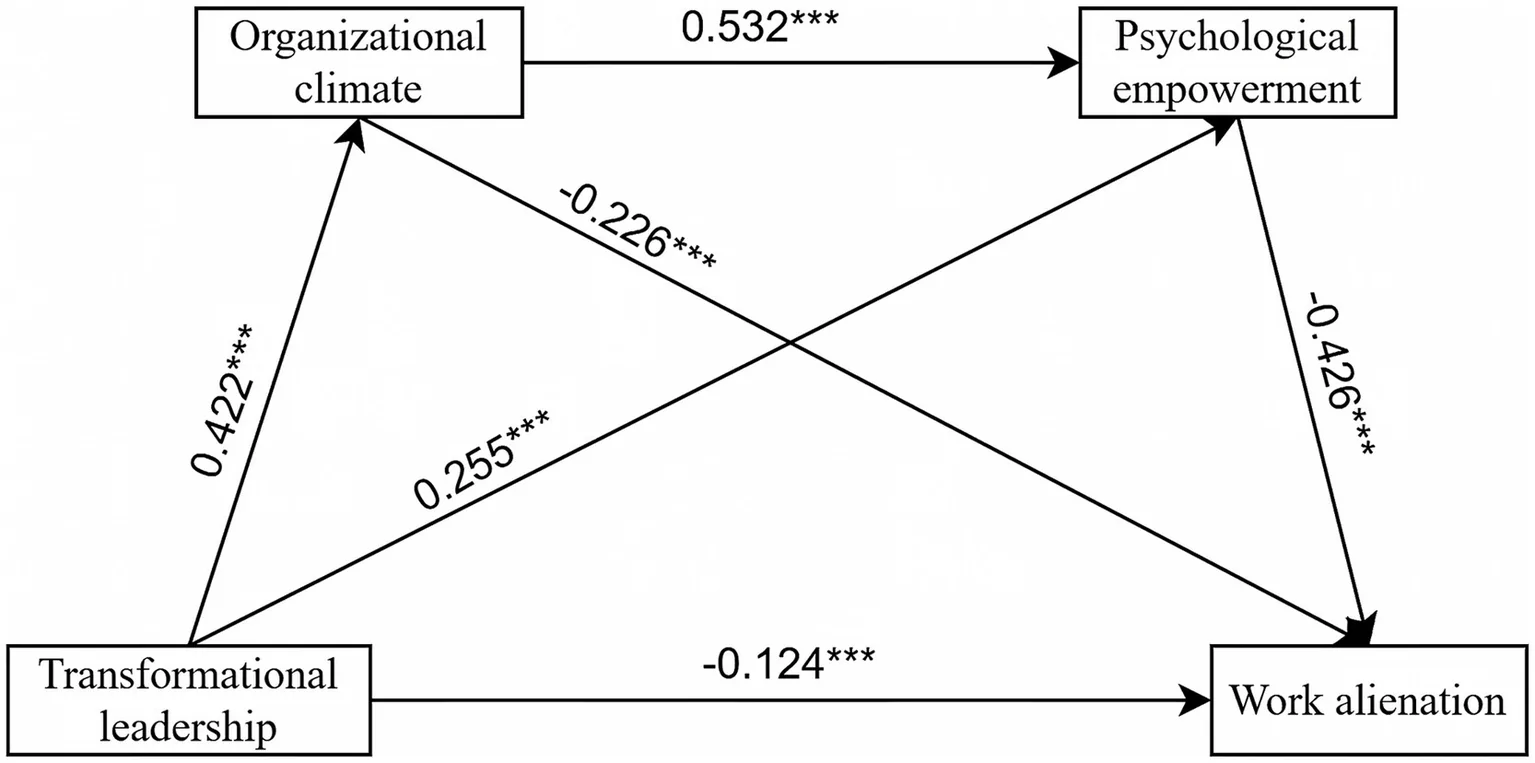
Chain mediation model of organizational climate and psychological empowerment between transformational leadership and work alienation. ***p < 0.001.

Table 4 shows that organizational climate and psychological empowerment significantly mediate the link between transformational leadership and work alienation, as indicated by the 95% bootstrap confidence intervals that do not include zero. Transformational leadership had a total effect of −0.4296 on nurses’ work alienation, with a direct effect of −0.1259, which constituted 29.3% of the total effect. The path from transformational leadership to work alienation through organizational climate had an effect of −0.0965, contributing 22.5% to the total effect. Meanwhile, the path through psychological empowerment had an effect of −0.1103, accounting for 25.6% of the total effect. Additionally, the combined path through both organizational climate and psychological empowerment resulted in an effect of −0.0969, comprising 22.6% of the total effect. Figure 1 visually illustrates the connections among transformational leadership, organizational climate, psychological empowerment, and work alienation.

**Table 4:** Chain mediating effect on transformational leadership, organizational climate, psychological empowerment, and work alienation (*N* = 1,154).

Effect type	Paths	Effect	BootSE	Boot 95% CI		Relative effect
				Lower	Upper	
Total effect		−0.4296	0.0268	−0.4822	−0.3770	100.0%
Direct effect	Transformational Leadership → Work Alienation	−0.1259	0.0255	−0.1758	−0.0760	29.3%
Indirect effect	Transformational Leadership → Organizational Climate → Work Alienation	−0.0965	0.0139	−0.1249	−0.0703	22.5%
	Transformational Leadership → Psychological Empowerment → Work Alienation	−0.1103	0.0129	−0.1359	−0.0859	25.6%
	Transformational Leadership → Organizational Climate → Psychological Empowerment → Work Alienation	−0.0969	0.0100	−0.1169	−0.0783	22.6%

## 5 Discussion

### 5.1 Interpretation of main findings

This study investigated work alienation among nurses in specific regions of Shandong Province, China. The results showed a mean work alienation score of 34.15 ± 11.42, indicating a moderate level. A systematic review (You et al., 2022) reported a score of 35.43 for Chinese nurses, which is slightly higher but consistent with our findings. These results suggest that work alienation is common among nurses and remains a concern, underscoring the need for nursing managers to address this issue.

Few studies have investigated the connection between transformational leadership and nurses’ work alienation. This study explored this relationship, revealing that nurses’ perceptions of their managers’ transformational leadership were directly and negatively correlated with their work alienation, thus supporting Hypothesis 1. This finding aligns with social exchange theory (Cropanzano and Mitchell, 2005), which suggests that high-quality leader-member exchanges promote reciprocity. When head nurses demonstrate transformational behaviors, nurses perceive this as organizational support, leading to increased commitment and reduced psychological detachment. Specifically, transformational leaders inspire subordinates through personal charisma, consideration, and intellectual stimulation (Bass, 1990), fulfilling nurses’ higher-order needs for self-actualization and professional growth. This fulfillment is crucial because work alienation often arises from unmet needs (Amarat et al., 2019). When nurses’ needs for recognition, autonomy, and meaningful work are met through transformational leadership, they are less likely to experience the helplessness and meaninglessness associated with work alienation. However, our direct effect was smaller than that reported in general employee populations (Boora and Singh, 2020). This discrepancy may be due to the hierarchical structure and high emotional labor demands in Chinese nursing settings, which might diminish the direct impact of leadership on alienation. This study extends social exchange theory by showing that the reciprocity triggered by transformational leadership in high power-distance contexts satisfies nurses’ needs not only through transactional support but also through identity-affirming recognition, suggesting that the theory’s assumption of instrumental exchange may require cultural qualification.

This study confirmed that organizational climate mediates the relationship between transformational leadership and work alienation, supporting Hypothesis 2. This finding aligns with social cognitive theory (Bandura, 1986), which posits that environmental cues influence individual cognitions and behaviors. Transformational leaders foster a positive organizational climate through shared visions and supportive team dynamics (Zhang et al., 2025), enhancing nurses’ psychological safety and reducing alienation. However, the mechanism by which organizational climate reduces work alienation may differ from its role in promoting positive outcomes. Zhang et al. (2025) showed that organizational climate mediates the relationship between transformational leadership and work engagement among ICU nurses in China, with the indirect effect accounting for 38.86% of the total effect. In our study, organizational climate mediates the reduction of work alienation, a negative outcome, rather than enhancing work engagement. This suggests that social cognitive theory’s focus on environmental cues shaping behavior may need to be supplemented with a stressor-reduction perspective when explaining the climate-alienation link. Moreover, our findings challenge the assumption that organizational climate mediates leadership effects uniformly across cultural contexts. In the high power-distance context of Chinese hospitals, nurses have limited formal avenues to influence organizational policies, making the climate created by the head nurse the primary environmental cue signaling organizational support. This cultural contingency extends social cognitive theory by demonstrating that the strength of environmental mediation depends not only on the quality of the climate but also on the structural availability of alternative environmental cues within the organization. Practically, nursing managers should focus on tangible climate improvements, such as fair scheduling, recognition systems, and open communication channels, to maximize the impact of their transformational behaviors.

Transformational leadership may indirectly reduce work alienation by enhancing psychological empowerment, supporting Hypothesis 3. Previous studies show a positive link between transformational leadership and psychological empowerment, which inversely relates to work alienation (Huang et al., 2025; Ibrahim et al., 2024; Li and Li, 2025; Zheng et al., 2026) his mediating pathway aligns with self-determination theory (Deci and Ryan, 1985), which posits that intrinsic motivation thrives when autonomy, competence, and relatedness are fulfilled. Transformational leaders meet these needs by offering individualized consideration, intellectual stimulation, and inspirational motivation (Ibrahim et al., 2024). When nurses are autonomous in decision making, competent in skills and connected through supportive relationships, their psychological empowerment grows. The empowerment shifts motivation from external regulation (working out of obligation) to identified regulation (working because of valuing outcomes) and integrated regulation (working because of identity). This shifts motivation from external regulation (working out of obligation) to identified regulation (working because of identity) and integrated regulation (working because of identity). This shift helps to understand work alienation, which is external regulation (working out of obligation, viewing work as imposed and meaningless) (Kanungo, 1982). However, indirect effects in our study were small compared to other healthcare settings. Ibrahim et al. (2024) found that psychological empowerment mediated almost fully the effect of transformational leadership on alienation-related outcomes for Saudi nurses, while direct effects were still significant. This suggests that for Chinese nurses psychological empowerment alone may not fully counteract work alienation due to high workloads and strained doctor-patient relationships, which create structural barriers that leadership-level empowerment cannot totally overcome. This challenges the assumption that intrinsic motivation arises naturally once basic needs are met. In constrained healthcare settings, need satisfaction may be necessary but not sufficient to reduce alienation. We contribute to self-determination theory by showing that psychological empowerment mediates leadership-alienation relationship and highlights the limitations of self-determination theory in uncertain occupational contexts.

Our findings reveal that transformational leadership influences nurses’ work alienation through the mediating roles of organizational climate and psychological empowerment, thus supporting Hypothesis 4. The chain mediation effect of −0.0969, which constitutes 22.6% of the total effect, aligns with insights from social cognitive theory and self-determination theory. Transformational leaders establish an organizational climate that signals support and fairness. These environmental cues subsequently shape individual cognitive evaluations, enhancing psychological empowerment and ultimately reducing work alienation.

The modest magnitude of this chain effect requires careful scrutiny. While previous studies have treated organizational climate and psychological empowerment as separate mediators, this study advances the literature by demonstrating their sequential interdependence. However, the remaining 77.4% of the effect, attributed to direct and individual paths, indicates that work alienation is influenced by multiple mechanisms operating concurrently. The sequential pathway is just one of several active processes. This pattern aligns with the complex etiology of work alienation in nursing, where structural constraints may limit the effectiveness of purely psychological pathways. Theoretically, this finding extends previous research by showing that leadership-alienation relationships involve multi-stage psychological processes. It also highlights that such sequential mechanisms account for only a minority of the total effect in structurally constrained healthcare environments.

In practice, nursing management should adopt a comprehensive approach that targets both organizational climate optimization and psychological empowerment, rather than focusing solely on transformational leadership training. These integrated strategies can more effectively reduce work alienation by engaging both environmental and individual psychological pathways, especially when direct interventions face organizational constraints.

### 5.2 Theoretical contributions

This study offers several theoretical contributions. First, it extends social exchange theory, social cognitive theory, and self-determination theory to the nursing field by empirically validating their explanatory power for work alienation. Second, it enhances understanding of leadership-alienation relationships by identifying a sequential mediation mechanism involving organizational climate and psychological empowerment, surpassing simple direct or single-mediator models. Third, it provides culturally specific evidence from the Chinese healthcare system, where hierarchical structures and high emotional labor demands may alter theoretical predictions based on Western samples. Fourth, by revealing that approximately 77.4% of the effect occurs through direct and individual mediating pathways, this study underscores the complexity of leadership-alienation relationships and the necessity for multi-theoretical frameworks to fully understand these dynamics.

### 5.3 Practical implications

The findings have significant implications for nursing management practices. First, hospital administrators should invest in transformational leadership training for head nurses, focusing on individualized consideration and intellectual stimulation. Second, interventions should aim to systematically enhance the organizational climate through fair scheduling, transparent communication, and recognition systems. Nursing managers must understand that organizational climate is a vital link between leadership behaviors and nurse outcomes. Third, psychological empowerment can be strengthened by granting nurses more autonomy in clinical decision-making and offering structured professional development opportunities. Finally, the sequential nature of the mediation indicates that climate improvement should either precede or accompany empowerment initiatives for maximum effectiveness. When nurses feel psychologically empowered, they are more likely to find their work meaningful and believe in their ability to complete tasks effectively, ultimately enhancing performance and reducing alienation.

### 5.4 Limitations and future directions

The present study has several limitations. First, its cross-sectional design limits the ability to infer causal relationships among variables. Although directional hypotheses were based on theoretical reasoning, measuring all variables simultaneously complicates ruling out reverse causation or common method bias. Future research should use longitudinal designs or experimental methods to verify causal pathways rigorously. Second, the data relied solely on nurses’ self-reports, potentially introducing common method bias. Despite applying Harman’s single-factor test for statistical control, this method effect cannot be entirely eliminated. Future studies should incorporate diverse data sources, such as manager and colleague evaluations or objective performance indicators. Third, the sample was drawn from healthcare institutions in a single region, limiting generalizability. Management systems and cultural backgrounds may vary across regions and hospital levels. Future research should replicate and validate the model in multicenter, cross-regional samples. Fourth, future studies could examine potential moderators like nurse tenure, hospital type, or specialty unit, as these may influence the strength of observed relationships. Finally, future research should explore whether the sequential mediation model identified here generalizes to other cultural contexts or healthcare systems.

## 6 Conclusion

This study discovered that organizational climate and psychological empowerment act as chain mediators between transformational leadership and nurses’ work alienation. Transformational leadership was directly linked to work alienation and indirectly through these mediators. These results underscore the various pathways by which transformational leadership influences work alienation. Therefore, nursing managers should focus on motivating and empowering individual nurses and enhancing the organizational climate.

## Data Availability

The raw data supporting the conclusions of this article will be made available by the authors, without undue reservation.
